# Stagnation in old age mortality among Finnish women: cause-of-death decomposition of life expectancy trends by income

**DOI:** 10.1177/14034948241266438

**Published:** 2024-08-21

**Authors:** Satu Malmberg, Lasse Tarkiainen, Liina Junna, Pekka Martikainen

**Affiliations:** 1 Helsinki Institute for Demography and Population Health, Helsinki, Finland; 2 Max Planck–University of Helsinki Center for Social Inequalities in Population Health, Helsinki, Finland; 3 Max Planck Institute for Demographic Research, Rostock, Germany

**Keywords:** Mortality, life expectancy, causes of death

## Abstract

**Aims:**

The decline in old age mortality and subsequent increase in life expectancy among older women has stalled in some high-income countries. The contribution of causes of death to and sub-group variations in these trends are generally not well understood. We assess trends in mortality and cause-of-death decomposition of life expectancy by income over the past 30 years in Finland.

**Methods:**

We obtained total population, annual register-based data on individuals (aged 30–89 years) residing in Finland in 1991–2020. We examined the trends in age-specific mortality rates and decomposed the contribution of various causes of death to changes in partial life expectancy among women aged 65–79 years over time and within each income quintile. In addition, we estimated life expectancy trends for the total population and by income quintile with and without causes related to alcohol consumption and smoking.

**Results:**

Our results indicate stagnation in mortality development among women in Finland aged 65–79 years. The slowdown of improvements in circulatory and heart disease mortality contributed substantially to the observed stagnation, although similar trends were observed in virtually all the causes of death we studied. The lowest income groups experienced the most adverse developments during the study period.

**Conclusions:**

**The stagnating life expectancy observed among Finnish women cannot be attributed solely to one cause of death. In contrast to findings on the topic from many other developed countries, smoking-related causes of death were of little significance. The stagnation is linked to growing inequality in mortality development among older women in Finland, which affects the overall trend.**

## Introduction

Stagnation in life expectancy among older women is observed in many, but not all, high-income countries. Stagnation or slowing down of mortality improvements has been observed in Denmark, the USA, Wales, England, Ireland, Taiwan and The Netherlands [1–8]. A significant body of literature on mortality stagnation among older women focuses on Denmark, where it has been quite prominent and was already observable in the 1990s [4, 7, 9–15]. However, some studies also suggest that the life expectancy of older Danish women is on the rise again [15, 16].

Comparative studies among countries with and without stagnating life expectancy indicate that causes related to smoking may be major contributors to the stagnation [6, 7, 13]. According to other findings, there is a slowdown in improvements in circulatory disease mortality in countries where stagnation is occurring [5, 8, 11]. Other potential causes mentioned include neurodegenerative diseases [5]. Mortality linked to diabetes and obesity has also been identified as one of the possible explanations [8]. We should point out that smoking may have an impact on circulatory diseases, several cancers and a range of other causes of death, which might not have been accounted for in all previous studies if the focus was on selected causes of death strongly related to smoking.

It has also been suggested that one of the factors driving life expectancy stagnation relates to social disparities in old age mortality whereby the trends in mortality outcomes are least favourable among the lowest socioeconomic groups [8–11]. Similar observations have also been made among younger women of working age in Denmark [17] and the UK [18], for example. Part of this development is attributable to socioeconomic differences in smoking, but alcohol-related deaths are also influential in explaining mortality development in deprived groups [19, 20]. However, there are variations among countries in terms of which socioeconomic groups are facing the most mortality stagnation [8, 9, 11, 17].

The aims of this study were: (a) to explore whether stagnation in old age mortality and life expectancy is observed among women in Finland; (b) to find out which causes of death contribute most to the potential stagnation, with special attention to causes related to smoking and alcohol use; and (c) to analyse the causes of death behind mortality development across income groups.

## Methods

We obtained annual register-based data for the entire population of Finland from Statistics Finland. Population and death registers were linked with the registers of the Finnish Tax Administration and the Social Insurance Institution using the encrypted personal identification code assigned to all permanent residents, and the Statistics Finland Board of Ethics has approved the register linkage (TK-53-1490-18). The participants were not contacted for consent following the national and international laws and guidelines concerning the use of register data originally collected for administrative and statistical purposes for scientific research: the Finnish Personal Data Act (523/1999), the Act on Secondary Use of Social and Healthcare Data (552/2019), the Finnish Statistics Act (280/2004) and the EU General Data Protection Regulation (GDPR).

We included all women aged 30–89 years between the years 1991 and 2020. We censored the individuals at death, end of follow-up or at the end of the year of their emigration. The income data were based on yearly household income subject to state taxation (including earned income, capital income and taxable benefits), divided by the square root of the total number of household members to account for economies of scale [21]. We calculated the income quintile cut-off points among the study subjects for each year separately within the studied age bracket.

To determine whether there were signs of mortality stagnation, we first created a contour plot of the differences in age-specific mortality rates using 2-year periods to smooth out fluctuations. First, we included both men and women aged 30–89 years. We then restricted the subsequent analysis to 65–79-year-old women (*n*=1,238,682) because the mortality rates showed signs of stagnation among women in this age interval, and because similar age restrictions have been utilised in previous studies on the topic [5–7]. Moreover, the age restriction helped to stabilise the income distribution for the remaining analyses, in which we addressed the income groups separately. The incomes of people below the age of 65 years may fluctuate strongly over time due to unemployment, sickness or parental leave, or unstable incomes among entrepreneurs, whereas most people over 65 years receive a more stable retirement income. We also calculated partial life expectancy [22] among women and men between the ages of 65 and 79 years for each year so that we could better establish and quantify the stagnation observed in the contour plots, and assess whether mortality stagnation was also occurring among men. Partial life expectancy refers to the expected number of years lived in an age interval; in the context of this study between ages 65 and 79 years, the maximum in the age interval being 15 years.

To assess which causes of death were leading the stagnation, we decomposed the change in partial life expectancy between each consecutive 5-year period in 1991–2020, as well as the contribution of the selected causes, using the Arriaga approach [22]. We pooled the yearly data within each period. The causes of death were classified according to the International Statistical Classification of Diseases, 10th revision, with harmonised coding for the preceding 9th revision. We categorised the causes based on the underlying cause as follows: ischaemic heart disease (I20–I25); cerebrovascular disease (I60–I69); other diseases of the circulatory system (I00–I15, I26–I28, I70–I99); dementia and Alzheimer’s disease (F01, F03, G30, R54); breast cancer (C50); lung cancer (C32–C34); other cancers (C00–C31, C35–C49, C51–C97); diseases of the respiratory system (J00–J64, J66–J99); accidents and violence (V01–X44, X46–Y89, U129); alcohol-related causes (F10, G312, G4051, G621, G721, I426, K292, K70, K860, K8600, O354, P043, X45); and all other causes. We further decomposed the changes in partial life expectancy for each income quintile from 1996–2000 to 2006–2010, and from 2006–2010 to 2016–2020.

In addition, to assess the impact of smoking and alcohol-related causes of death more specifically, we estimated life expectancy between the ages of 65 and 79 years for 5-year periods with and without causes related to smoking and alcohol, for all women and for each income quintile, in addition to cases in which alcohol-related deaths were defined as above. We used the same categories of underlying causes, and also included cases in which any of the same alcohol-related causes were reported on the death certificate as a contributing cause. This approach provides a more comprehensive picture of the contribution of alcohol to mortality [23].

In assessing the impact of smoking on mortality we applied the method developed by Preston et al. (2010) [24]. Accordingly, we used age and sex-specific lung cancer death rates as an indicator of damage from smoking, and introduced a regression model based on lung cancer mortality in 21 high-income countries among those aged 50 years and above to predict mortality from other causes of death. This model can be employed to evaluate the proportion of total deaths attributable to smoking [24].

## Results

Finnish women aged between 60 and 79 years experienced stagnation in mortality that started during the late 2000s (Supplemental Figures 1 and 2). Age groups below 60 years, in contrast, continued to display fluctuating but generally declining mortality in relative terms, but also in terms of absolute age-specific mortality. Male mortality follows a similar pattern, but the negative development is not as pronounced (Supplemental Figures 3 and 4).

**Figure 1.**
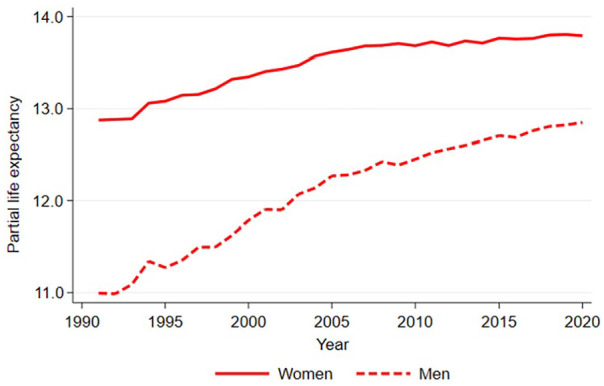
Partial life expectancy between ages 65 and 79 years among women and men in Finland in 1991–2020.

Figure 1 shows partial life expectancy trends at ages 65–79 years (a maximum of 15 years in the age interval) and indicates a plateau in women’s partial life expectancy improvement from 2005 onwards. The rate of improvement among women had not shown signs of recovery by 2020. In contrast, men’s life expectancy continued to increase and has not shown noticeable stagnation. However, the rate of improvement among men slowed somewhat after the late 2000s compared with previous years.

**Figure 2.**
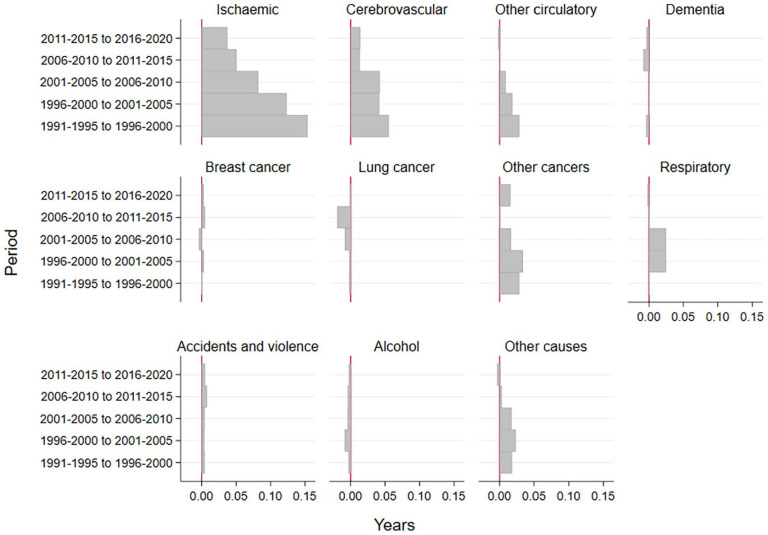
The contribution of causes of death to the change in partial life expectancy between ages 65 and 79 years among women in Finland for 5-year periods in 1991–2020.

The reduction in mortality to ischaemic heart disease, cerebrovascular disease and other circulatory diseases slowed down markedly across the study periods (Figure 2). Moreover, slowed-down reduction or adverse development was visible in almost all causes of death among women in this age interval.

**Figure 3.**
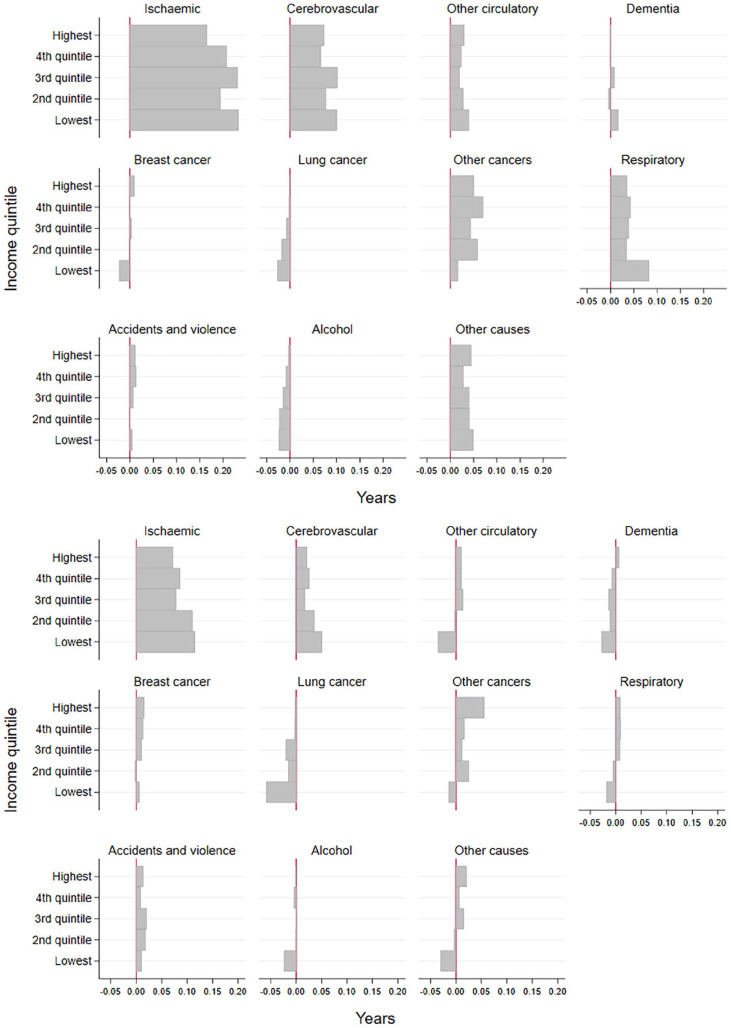
Cause-specific contributions to changes in partial life expectancy between ages 65 and 79 years by income quintiles among women in Finland from 1996–2000 to 2006–2010 (a), and from 2006–2010 to 2016–2020 (b).

The plateau in women’s partial life expectancy at ages 65–79 years was particularly visible among those in the lowest income quintile (Supplemental Figure 5). This was due to slowed-down reduction in mortality or adverse development in multiple causes of death. Reductions in mortality to ischaemic heart disease, cerebrovascular disease and other circulatory diseases slowed down notably in all income groups in 2006–2020 compared with 1996–2010 (Figure 3(a) and (b)). However, although the lowest income quintile experienced the biggest reduction in mortality attributable to ischaemic and cerebrovascular diseases among the income groups in the later period, they also experienced an increase in mortality attributable to other circulatory diseases Figure 3(b). Mortality attributable to lung cancer also increased markedly in the lowest income quintile in 2006–2010; in addition, dementia, other cancers, respiratory diseases and alcohol-related and other causes of death showed a more negative development compared with the higher income quintiles. The only notable positive development in the second period was among the highest income quintiles and concerned mortality to other cancers.

**Figure 4.**
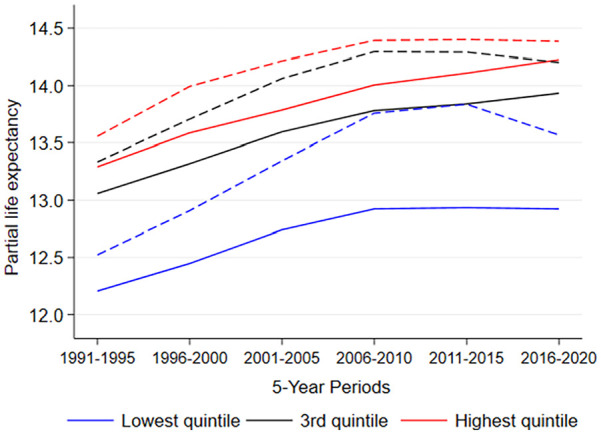
Partial life expectancy between ages 65 and 79 years by income quintiles with (solid line) and without (dashed line) alcohol and smoking-related deaths among women in Finland (1991–2020).

Smoking and alcohol-related deaths reduced life expectancy across all income quintiles (Figure 4, for full results see Supplemental Figure 6). All income quintiles gained in life expectancy after 2006–2010, albeit with a slower rate of increase than previously except for the lowest quintile in which it stalled completely.

The combined impact of smoking and alcohol-related causes on women’s life expectancy first increased in 1991–2010 (Supplemental Figure 7). However, the combined impact of these causes then decreased sharply between the 5-year periods examined later, namely 2011–2015 and 2016–2020. This development is particularly interesting in that life expectancy continued to stall in 2011–2020. Excluding alcohol and smoking-related deaths, the increase in life expectancy would have been around 0.5 years higher in 2006–2010 and around 0.3 years in 2016–2020. Although alcohol alone as well as alcohol and smoking-related causes in combination had a diminishing influence on life expectancy, the impact of smoking-related causes alone increased slightly after 2006–2010. In other words, smoking- and alcohol-related deaths seemed to lose some of their power in explaining the life expectancy changes during the last period (2016–2020) among all income quintiles (Figure 4, Supplemental Figures 6 and 8) and thus life expectancy with and without alcohol and smoking-related causes started to converge. In addition, while the differences in life expectancy between the highest and lowest income quintiles has grown since the 1990s, the role of smoking and alcohol-related deaths in the increasing gap has been declining since the 2006–2010 period (Figure 4, Supplemental Figure 9).

## Discussion

### Main findings

The results reveal clear stagnation in mortality rates and life expectancy among older women (ages 65–79 years) in Finland. This slowing pace of mortality improvement is evident across all except the highest income quintile. The economically most disadvantaged are experiencing the most adverse developments.

In general, smoking-related causes were not the main factor driving old age mortality stagnation in Finland, and the combined effect of smoking and alcohol-related causes on changes in life expectancy has been decreasing since 2010. The slowdown in improvements in mortality attributable to circulatory and heart disease was responsible for much of the stagnation. However, it appears that no single group of causes can be blamed, and that the stagnation has more to do with an overall stalling in mortality improvement, particularly in lower income groups.

### Causes of death on the population level

In contrast to findings reported in numerous studies from other countries, such as those conducted in Denmark [12–14] and comparison studies involving the USA, The Netherlands, Japan and France [6, 7], smoking-related causes are not the main driving force behind the stagnation in women’s mortality in Finland, although they are slowly becoming more influential. The relatively modest contribution of smoking to mortality stagnation by socioeconomic status (SES) at the ages observed in this study may relate to the late initiation of smoking among Finnish women, the relatively low prevalence of smoking altogether and – by international standards – the modest SES differences in smoking among women [20].

Another health-related behaviour that has had a strong effect on fluctuations in male mortality in Finland is alcohol consumption [19, 20, 25]. Among women, however, the negative impact of alcohol-related deaths on life expectancy strengthened until 2006–2010 but declined swiftly after that. This accords with general population-level trends in alcohol-related mortality in Finland, explaining the diminishing role of alcohol in life expectancy among women [26]. Instead, we found that setbacks in mortality attributable to circulatory and heart disease were largely responsible, which is consistent with findings reported in studies by Djeundje et al. [8] and Meslé and Vallin [5] covering several economically developed countries and study periods from the 1950s until 2017.

The stagnation in mortality attributable to circulatory and heart diseases could be connected to the finding that obesity is becoming more common among older women [8]. Our data did not allow for directly studying any connection between the setbacks in mortality and increased obesity, which is a point to consider in future research. However, health surveys and health monitoring carried out in Finland indicate that obesity among women was increasing throughout 2000–2020 [27, 28], thus making it a credible candidate as a factor contributing to the stagnation in mortality.

### Inequalities in life expectancy according to income

Our findings support the notion that inequality in partial life expectancy among women is growing. In addition, the adverse development in the lower income groups is reflected in the overall stagnation of life expectancy at ages 65–79 years, as observed elsewhere [8, 10, 11]. Similar results have also been found for other indicators of socioeconomic position, such as education [16, 29]. We observed that partial life expectancy in the lowest income quintile began to stall after 2006, that middle income groups experienced a slowing pace of improvement in life expectancy, and that life expectancy in the highest group continued to rise in an upward trajectory almost as steeply as before. This finding was different from what was observed in Denmark in the 1990s, as the groups experiencing the strongest stagnation in life expectancy comprised the low middle and middle affluence groups and not the group with the lowest income [11]. However, there is some evidence that women with low educational attainment are the ones who have been left behind in terms of mortality improvement in Denmark [17]. We cannot directly assess whether the adverse impact of low income on survival has strengthened, or whether people with poorer health have become more likely to be selected into lower socioeconomic positions. Neither can we directly assess whether unhealthy behaviours are increasingly prevalent in lower income groups, or whether deteriorating health is attributable to suffering worse consequences from similar behaviours compared with wealthier peers. Our results indicate that alcohol consumption and smoking are unlikely to be major factors. However, obesity is more prevalent and is increasing more rapidly in lower socioeconomic groups in Finland [27].

### Methodological considerations

The study is based on high-quality register data, which is also free of self-report bias. It covers the entire Finnish population over three decades, with no loss to follow-up.

Using contributory causes of death, we were able to estimate overall alcohol-attributable mortality levels more accurately than if we had only considered the underlying cause [23]. In addition to assessing the role of lung cancer, we utilised an indirect method to estimate smoking-related mortality, avoiding issues related to self-reported smoking data such as recall bias and preferential reporting. Unlike approaches adopted in observational cohort studies, this one considers lifetime smoking exposure, including factors such as inhalation and exposure to passive smoking. It also minimises problems associated with single-time measurements of smoking.

This study concerns household income, which is an indicator of economic resources and living standards. The measure is particularly useful among older women, as many women who have lower incomes may still be financially very comfortable due to a high earning partner and pooling of income in the household. Future studies are needed to address other measures of socioeconomic position such as individual income, occupational class, or wealth.

In interpreting our findings, we acknowledge the possibility of a ceiling effect given our focus on partial life expectancy between ages 65 and 79 years, with a maximum expectancy of 15 years. However, the stagnation we observed cannot be entirely attributed to this factor because we observed increased mortality rates in many causes of death across various income groups, and we saw no ceiling effects among the highest earners, who also have the highest life expectancy. The indications are that other significant factors contributed to the stagnation we observed, and that the ceiling effect is not the explanation. The latest year in our study period was marked by the unforeseen global COVID-19 pandemic that potentially could have affected our results. However, given that only 47 women aged between 65 and 79 years died directly of COVID-19 in 2020 [30], it is unlikely that the pandemic strongly influenced our results. Future research is needed to quantify the magnitude of the effect of COVID-19 on mortality trends at these ages.

## Conclusions

We observed stagnation in mortality development among older women in Finland and found that it cannot be attributed solely to one cause of death, although slowed reductions in circulatory and heart disease mortality are major contributors. Our results support the previous finding that stagnation in life expectancy occurs particularly in the lowest income groups, which affects the overall trend. Alcohol and smoking-related causes are not major contributors to the stagnation, but the effect of smoking has been slightly increasing in more recent years and may increase further in years to come.

## Supplemental Material

sj-docx-1-sjp-10.1177_14034948241266438 – Supplemental material for Stagnation in old age mortality among Finnish women: cause-of-death decomposition of life expectancy trends by income Supplemental material, sj-docx-1-sjp-10.1177_14034948241266438 for Stagnation in old age mortality among Finnish women: cause-of-death decomposition of life expectancy trends by income by Satu Malmberg, Lasse Tarkiainen, Liina Junna and Pekka Martikainen in Scandinavian Journal of Public Health

## References

[1] Freedman VA Wolf DA Spillman BC. Disability-free life expectancy over 30 years: a growing female disadvantage in the US population. Am J Public Health 2016;106:1079–1085. 26985619 10.2105/AJPH.2016.303089 PMC4860065

[2] Hiam L Harrison D McKee M , et al. Why is life expectancy in England and Wales ‘stalling’? J Epidemiol Community Health 2018;72:404–408. 29463599 10.1136/jech-2017-210401

[3] Leon DA Jdanov DA Shkolnikov VM. Trends in life expectancy and age-specific mortality in England and Wales, 1970–2016, in comparison with a set of 22 high-income countries: an analysis of vital statistics data. Lancet Public Health 2019;4:e575–e582. 10.1016/S2468-2667(19)30177-X 31677776

[4] Lindahl-Jacobsen R Keiding N Lynge E. Long term mortality trends behind low life expectancy of Danish women. J Epidemiol Community Health 2002;56:205–208. 11854342 10.1136/jech.56.3.205 PMC1732096

[5] Meslé F Vallin J. Diverging trends in female old-age mortality: the United States and the Netherlands versus France and Japan. Popul Dev Rev 2006;32:123–145.

[6] Rostron BL Wilmoth JR. Estimating the effect of smoking on slowdowns in mortality declines in developed countries. Demography 2011;48:461–479. 21519979 10.1007/s13524-011-0020-9

[7] Staetsky L. Diverging trends in female old-age mortality: a reappraisal. Demogr Res 2009;21:885–914.

[8] Djeundje VB Haberman S Bajekal M , et al. The slowdown in mortality improvement rates 2011–2017: a multi-country analysis. Eur Actuar J 2022;12:839–878.

[9] Kallestrup-Lamb M Kjærgaard S Rosenskjold CPT . Insight into stagnating adult life expectancy: analyzing cause of death patterns across socioeconomic groups. Health Econ 2020;29:1728–1743. 32969122 10.1002/hec.4166

[10] Blake D Kallestrup-Lamb M Kjaergaard S , et al. Insight into stagnating life expectancy: analysing cause of death patterns across socio-economic groups. London: Pensions Institute, 2020. 10.1002/hec.4166 32969122

[11] Kallestrup-Lamb M Rosenskjold CPT . Insight into the female longevity puzzle: using register data to analyse mortality and cause of death behaviour across socio-economic groups. Report. Aarhus, Denmark: CREATES Research Papers, 2017.

[12] Lindahl-Jacobsen R Keiding N Lynge E. Causes of death behind low life expectancy of Danish women. Scand J Public Health 2006;34:432–436. 16861194 10.1080/14034940500489842

[13] Lindahl-Jacobsen R Von Euler M Osler M , et al. Women’s death in Scandinavia – what makes Denmark different? Eur J Epidemiol 2004;19:117–121. 15074566 10.1023/b:ejep.0000017834.35943.bd

[14] Lindahl-Jacobsen R Oeppen J Rizzi S , et al. Why did Danish women’s life expectancy stagnate? The influence of interwar generations’ smoking behaviour. Eur J Epidemiol 2016;31:1207–1211. 27637782 10.1007/s10654-016-0198-7

[15] Lindahl-Jacobsen R Rau R Jeune B , et al. Rise, stagnation, and rise of Danish women’s life expectancy. Proc Natl Acad Sci 2016;113:4015–4020. 27035998 10.1073/pnas.1602783113 PMC4839462

[16] Brønnum-Hansen H Németh L Jasilionis D , et al. National and education-specific trends in life and health expectancies in Denmark 2004–2015. Scand J Public Health 2024;52:175–183. 36600445 10.1177/14034948221144348

[17] Brønnum-Hansen H Baadsgaard M. Increasing social inequality in life expectancy in Denmark. Eur J Public Health 2007;17:585–586. 17470463 10.1093/eurpub/ckm045

[18] Marshall L Finch D Cairncross L , et al. Mortality and life expectancy trends in the UK: Stalling progress. London: The Health Foundation, 2019.

[19] Martikainen P Mäkelä P Peltonen R , et al. Income differences in life expectancy: the changing contribution of harmful consumption of alcohol and smoking. Epidemiology 2014;25:182–190. 24487202 10.1097/EDE.0000000000000064

[20] Östergren O Martikainen P Tarkiainen L , et al. Contribution of smoking and alcohol consumption to income differences in life expectancy: evidence using Danish, Finnish, Norwegian and Swedish register data. J Epidemiol Community Health 2019;73:334–339. 30674585 10.1136/jech-2018-211640 PMC6581103

[21] OECD Project on Income Distribution and Poverty. What are equivalence scales? https://www.oecd.org/economy/growth/OECD-Note-EquivalenceScales.pdf (accessed 6 March 2024).

[22] Arriaga EE. Measuring and explaining the change in life expectancies. Demography 1984;21:83–96. 6714492

[23] Trias-Llimós S Martikainen P Mäkelä P , et al. Comparison of different approaches for estimating age-specific alcohol-attributable mortality: the cases of France and Finland. PloS One 2018;13:e0194478. 10.1371/journal.pone.0194478 PMC5864025 29566081

[24] Preston SH Glei DA Wilmoth JR. A new method for estimating smoking-attributable mortality in high-income countries. Int J Epidemiol 2010;39:430–438. 20032265 10.1093/ije/dyp360 PMC2915474

[25] Stickley A Baburin A Jasilionis D , et al. Economic cycles and inequalities in alcohol-related mortality in the Baltic countries and Finland in 2000–2015: a register-based study. Addiction 2021;116:3357–3368. 33908662 10.1111/add.15526

[26] Official Statistics of Finland (OSF). Causes of death. Number of deaths from alcohol on level with the year before. https://www.stat.fi/til/ksyyt/2020/ksyyt_2020_2021-12-10_kat_004_en.html (2021, accessed 6 March 2024).

[27] Kagenaar E Van Hemelrijck WMJ Kunst AE , et al. Long-term trends in obesity prevalence by socio-economic group in five European countries and the USA: the relevance of the diffusion of innovations theory. Obes Facts 2022;15:753–761. 36108604 10.1159/000527070 PMC9801347

[28] Finnish Institute for Health and Welfare. Yhä useampi työikäinen on ylipainoinen (More and more people of working age are overweight). https://thl.fi/-/yha-useampi-tyoikainen-on-ylipainoinen (2021, accessed 6 March 2024).

[29] Enroth L Jasilionis D Németh L , et al. Changes in socioeconomic differentials in old age life expectancy in four Nordic countries: the impact of educational expansion and education-specific mortality. Eur J Ageing 2022;19:161–173. 35663915 10.1007/s10433-022-00698-y PMC9156635

[30] Statistics Finland. Statistics Finland’s free-of-charge statistical databases: Deaths by underlying cause of death (time series classification), age and sex, 1969–2021. https://pxdata.stat.fi/PXWeb/pxweb/en/StatFin/StatFin__ksyyt/statfin_ksyyt_pxt_11az.px (2024, accessed 6 March 2024).

