# Supplementary material for: Stagnation in old age mortality among Finnish women: cause-of-death decomposition of life expectancy trends by income
Source: Scand J Public Health. 2024 Aug 21;53(7):756–63. doi: 10.1177/14034948241266438 (PMC12598063; doi:10.1177/14034948241266438)

**Supplementary Files**

**Supplementary Figure 1.** Trends in absolute differences in mortality rates between consecutive two-year periods in two-year age groups among women aged 30-89 in Finland (1991-2020) (positive values indicate mortality increase) ^a^

**
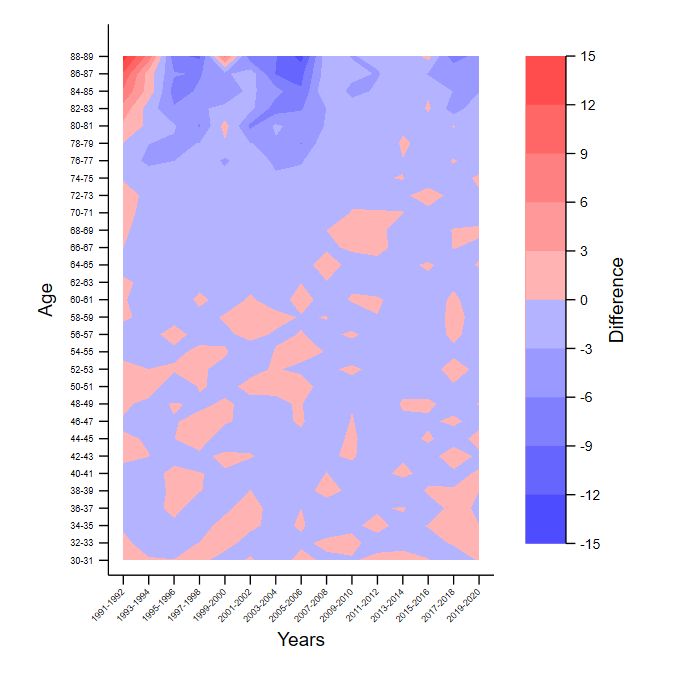
**

^a^ Absolute mortality rate difference was calculated as the differences between two time points = mortality at time t in age interval x – mortality at time t-1 in age interval x.

**Supplementary Figure 2.** Trends in relative differences in mortality rates between consecutive two-year periods in two-year age groups for women aged 30-89 in Finland (1991-2020) (positive values indicate mortality increase) ^a^


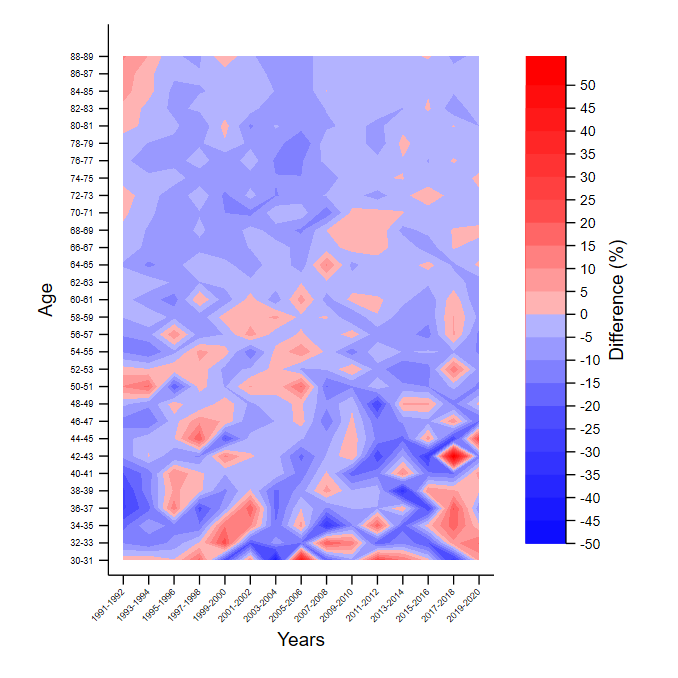


^a^ Relative difference was calculated by dividing the absolute difference in mortality (i.e. the change in mortality rate) with mortality at time t-1 and multiplying this by 100 to present the results as percentages.

**Supplementary Figure 3.** Trends in absolute differences in mortality rates between consecutive two-year periods in two-year age groups for men aged 30-89 in Finland (1991-2020) (positive values indicate mortality increase) ^a^

**
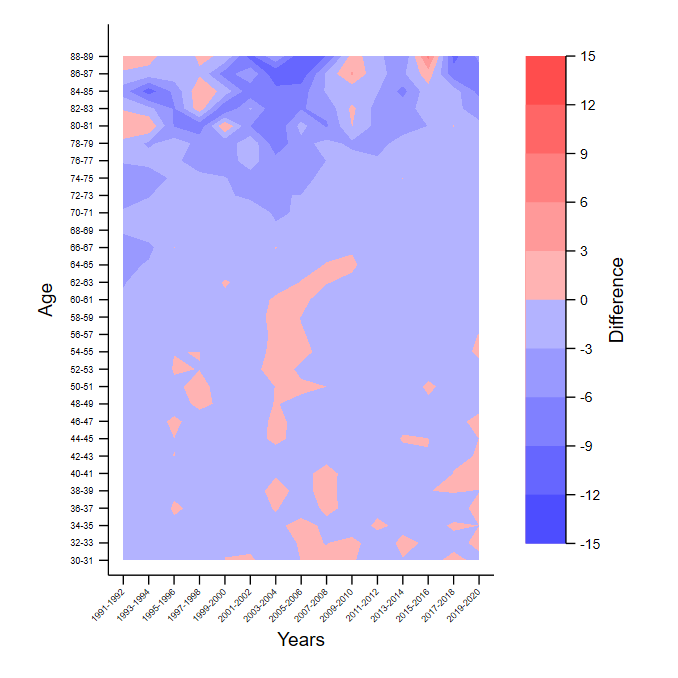
**

^a^ Absolute mortality rate difference was calculated as the differences between two time points = mortality at time t in age interval x – mortality at time t-1 in age interval x.

**Supplementary Figure 4.** Trends in relative differences in mortality rates between consecutive two-year periods in two-year age groups for men aged 30-89 in Finland (1991-2020) (positive values indicate mortality increase) ^a^

**
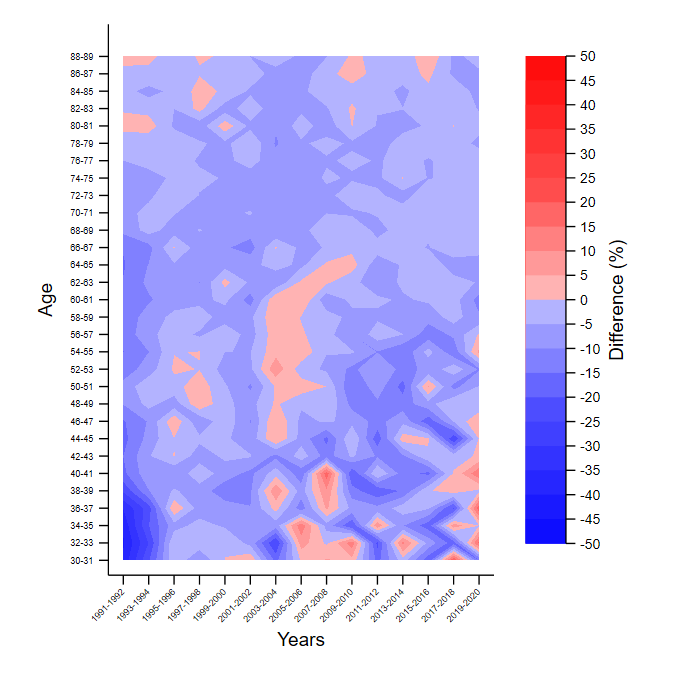
**

^a^ Relative difference was calculated by dividing the absolute difference in mortality (i.e. the change in mortality rate) with mortality at time t-1 and multiplying this by 100 to present the results as percentages.

**Supplementary Figure 5.** Partial life expectancy between ages 65 and 79 by income quintiles among women in Finland (1991-2020)


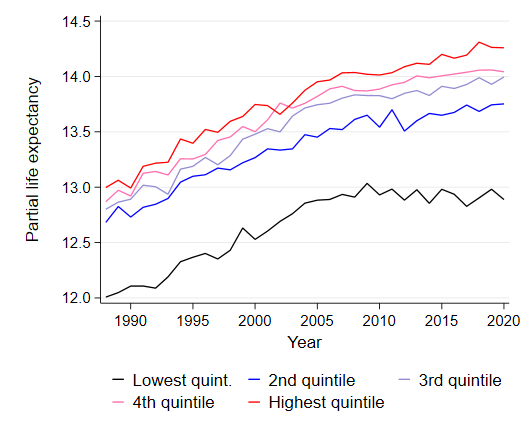


**Supplementary Figure 6.** Partial life expectancy between age 65 and 79 by income quintiles with (solid line) and without (dashed line) alcohol-and smoking-related deaths among women in Finland (1991-2020)

**
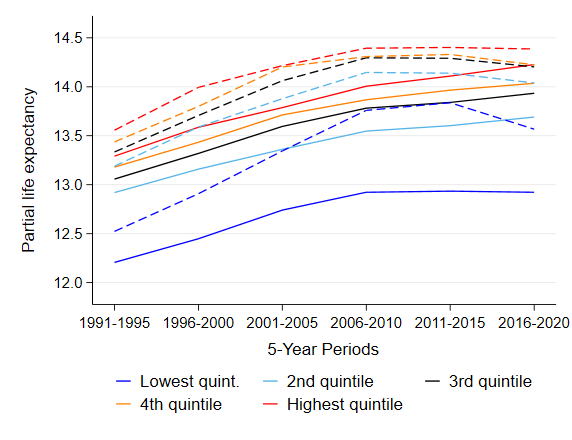
**

**Supplementary Figure 7.** Differences in partial life expectancy between ages 65 and 79 with and without alcohol- and smoking-related deaths among women in Finland (1991-2020)


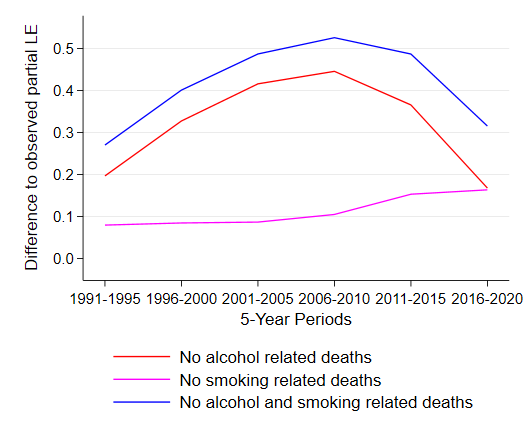


**Supplementary Figure 8.** Observed differences in partial life expectancy between ages 65 and 79 to the highest income quintile with and without (dashed line) smoking-related deaths


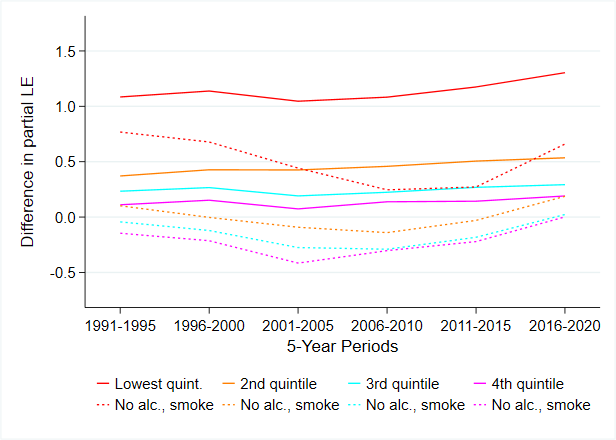


**Supplementary Figure 9****.** Differences in partial life expectancy between ages 65 and 79 among the highest vs. the lowest income quintiles when alcohol- and smoking-related deaths are excluded


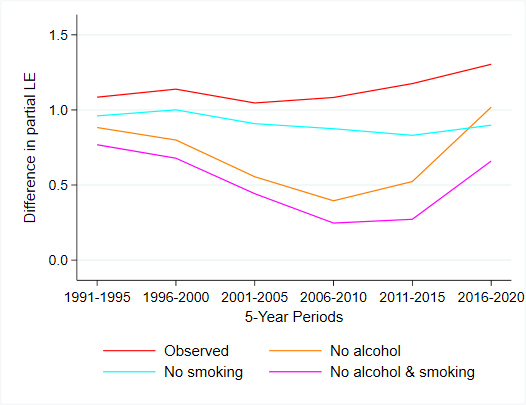

Supplement: sj-docx-1-sjp-10.1177_14034948241266438 – Supplemental material for Stagnation in old age mortality among Finnish women: cause-of-death decomposition of life expectancy trends by income [file sj-docx-1-sjp-10.1177_14034948241266438.docx]
